# Swine Genome Sequencing Consortium (SGSC): A Strategic Roadmap for Sequencing The Pig Genome

**DOI:** 10.1002/cfg.479

**Published:** 2005-06

**Authors:** Lawrence B. Schook, Jonathan E. Beever, Jane Rogers, Sean Humphray, Alan Archibald, Patrick Chardon, Denis Milan, Gary Rohrer, Kellye Eversole

**Affiliations:** 1 Institute for Genomic Biology University of Illinois Urbana IL USA; 2 Department of Animal Sciences University of Illinois Urbana IL USA; 3 The Wellcome Trust Sanger Institute Hinxton UK; 4 Roslin Institute Edinburgh UK; 5 INRA-CEA Jouy-en-Josas France; 6 INRA-Toulouse France; 7 Agricultural Research Service Clay Center NE USA; 8 The Alliance for Animal Genomics Bethesda MD USA; 9 Departments of Animal Sciences and Veterinary Pathobiology University of Illinois 382 Edward R. Madigan Laboratory 1201 W. Gregory Dr. Urbana IL 61801 USA

## Abstract

The Swine Genome Sequencing Consortium (SGSC) was formed in September 2003 by academic, government and industry representatives to provide international coordination for sequencing the pig genome. The SGSC’s mission is to advance biomedical research for animal production and health by the development of DNAbased tools and products resulting from the sequencing of the swine genome. During the past 2 years, the SGSC has met bi-annually to develop a strategic roadmap for creating the required scientific resources, to integrate existing physical maps, and to create a sequencing strategy that captured international participation and a broad funding base. During the past year, SGSC members have integrated their respective physical mapping data with the goal of creating a minimal tiling path (MTP) that will be used as the sequencing template. During the recent Plant and Animal Genome meeting (January 16, 2005 San Diego, CA), presentations demonstrated that a human–pig comparative map has been completed, BAC fingerprint contigs (FPC) for each of the autosomes and X chromosome have been constructed and that BAC end-sequencing has permitted, through BLAST analysis and RH-mapping, anchoring of the contigs. Thus, significant progress has been made towards the creation of a MTP. In addition, whole-genome (WG) shotgun libraries have been constructed and are currently being sequenced in various laboratories around the globe. Thus, a hybrid sequencing approach in which 3x coverage of BACs comprising the MTP and 3x of the WG-shotgun libraries will be used to develop a draft 6x coverage of the pig genome.


**Comparative and Functional Genomics**
*Comp Funct Genom* 2005; **6**: 251–255.
Published online in Wiley InterScience (www.interscience.wiley.com). **DOI:** 10.1002/cfg.479

**Conference Review**

### Swine Genome Sequencing Consortium (SGSC): a strategic roadmap for sequencing the pig genome

Lawrence B. Schook[1,2]*, Jonathan E. Beever[1,2], Jane Rogers[3], Sean Humphray[3], Alan Archibald[4], Patrick Chardon[5], Denis Milan[6], Gary Rohrer[7] and Kellye Eversole[8]

[1] *Institute for Genomic Biology, University of Illinois, Urbana, IL, USA*
[2] *Department of Animal Sciences, University of Illinois, Urbana, IL, USA*
[3] *The Wellcome Trust Sanger Institute, Hinxton, UK*
[4] *Roslin Institute, Edinburgh, UK*
[5] *INRA-CEA, Jouy-en-Josas, France*
[6] *INRA-Toulouse, France*
[7] *Agricultural Research Service, Clay Center, NE, USA*
[8] *The Alliance for Animal Genomics, Bethesda, MD, USA*

**Correspondence to: Lawrence B. Schook, Departments of Animal Sciences and Veterinary Pathobiology, University of Illinois, 382 Edward R. Madigan Laboratory, 1201 W. Gregory Dr., Urbana, IL 61801, USA. E-mail: schook@uiuc.edu*

Received: 22 February 2005
Revised: 17 March 2005
Accepted: 18 March 2005

### Abstract

**The Swine Genome Sequencing Consortium (SGSC) was formed in September 2003 by academic, government and industry representatives to provide international coordination for sequencing the pig genome. The SGSC's mission is to advance biomedical research for animal production and health by the development of DNA-based tools and products resulting from the sequencing of the swine genome. During the past 2 years, the SGSC has met bi-annually to develop a strategic roadmap for creating the required scientific resources, to integrate existing physical maps, and to create a sequencing strategy that captured international participation and a broad funding base. During the past year, SGSC members have integrated their respective physical mapping data with the goal of creating a minimal tiling path (MTP) that will be used as the sequencing template. During the recent Plant and Animal Genome meeting (January 16, 2005 San Diego, CA), presentations demonstrated that a human–pig comparative map has been completed, BAC fingerprint contigs (FPC) for each of the autosomes and X chromosome have been constructed and that BAC end-sequencing has permitted, through BLAST analysis and RH-mapping, anchoring of the contigs. Thus, significant progress has been made towards the creation of a MTP. In addition, whole-genome (WG) shotgun libraries have been constructed and are currently being sequenced in various laboratories around the globe. Thus, a hybrid sequencing approach in which 3× coverage of BACs comprising the MTP and 3× of the WG-shotgun libraries will be used to develop a draft 6× coverage of the pig genome.** **Copyright © 2005 John Wiley & Sons, Ltd.**

**Keywords:** genomics; pig genome; porcine; physical maps; sequencing consortium; whole-genome sequencing

### Background and discussion

The pig genome is of similar size, complexity and chromosomal organization ($2n = 38$, including meta- and acrocentric chromosomes) as the human genome. Over the past decade tremendous progress has been made in mapping and characterizing the swine genome. Currently, moderate- to high-resolution genetic linkage maps containing highly polymorphic loci (Type II) have been produced using independent mapping populations (Rohrer *et al.*, 1996; Ellegren *et al.*, 1994; Archibald *et al.*,

Copyright © 2005 John Wiley & Sons, Ltd.

1995). Additionally, physical mapping methods such as somatic cell hybrid analysis (Rettenberger *et al.*, 1994; Yerle *et al.*, 1996), in situ hybridization, and ZOO-FISH (Chowdhary *et al.*, 1996; Fronicke *et al.*, 1996; Goureau *et al.*, 1996) have been employed to enrich the Type I marker map, and to perform comparative analysis with map-rich species such as the human and mouse. To date, >3000 mapped loci are catalogued for the pig genome (**http://www.thearkdb.org**). Recently, whole-genome radiation hybrid (WG-RH) panels (7500 and 12 500 rad) have been generated for swine (Hawken *et al.*, 1999; Yerle *et al.*, 2002), resulting in yet another rapid increase in the number of expressed sequences being mapped, facilitating comparative mapping with other species (Rinke *et al.*, 2002). The swine genomics community has also acquired access to resources such as bacterial artificial chromosome (BAC) libraries (Fahrenkrug *et al.*, 2001; Anderson *et al.*, 2000) that provide approximately 35× coverage of the swine genome. These BAC resources have facilitated the production of high-resolution physical maps in specific chromosomal regions (Rogel-Gaillard *et al.*, 1999; Milan *et al.*, 2000) and support the construction of sequence-ready mapping resources for the porcine genome.

Comparative maps have indicated that the porcine and human genomes are more similarly organized than when either is compared to the mouse (Thomas *et al.*, 2003). The mean length of conserved syntenic segments between human and pig is approximately twice as long as the average length of conserved syntenic segments between human and mouse (Ellergren *et al.*, 1994; Rettenberger *et al.*, 1995). Furthermore, the organizational similarities between the human and porcine genomes are reflected in similarities at the nucleotide level. In more than 600 comparisons of non-coding DNAs aligned by orthologous exonic sequences on human chromosome 7, pig (and cow, cat and dog) sequences consistently grouped closer to human and non-human primate sequences than did rodent (mouse and rat) sequences (Thomas *et al.*, 2003). Furthermore, the rodent genomes are evolving at a different (faster) rate than other representative genomes. For these reasons it is necessary to produce the genomic sequence for eutherian mammals outside the primate and rodent lineages in order to better assemble and annotate the human sequence. During the Plant and Animal Genome meeting, it was reported that a 1.0 Mb human–pig comparative map has been completed (Meyers *et al.*, 2005). This map will provide the basis for creating a MTP that will be used as the template for genome sequencing.

### Harvesting genomic information

The porcine research community has a long history in quantitative genetics, and more recently in genomics research. The genetic contribution of many polygenic traits in pigs is well documented, and this knowledge has provided the basis for the identification and mapping of a growing number of quantitative trait loci (QTL) (Andersson *et al.*, 1994; Milan *et al.*, 2000; Rohrer *et al.*, 1999; Wilkie *et al.*, 2000; Paszek *et al.*, 2000; Malek *et al.*, 2001a,b; Nezer *et al.*, 2002). These maps have been used to identify chromosomal regions that influence quantitative traits affecting growth, body composition, reproduction and immune response (Bidanel and Rothschild, 2002). The quantitative trait loci defined in these studies often span 20–40 centiMorgans (cM) and perhaps correspond to about 20–40 Mbp of DNA. These initial scans for the gene(s) controlling the phenotype of interest generally only reduce the search space to 1–2% of the genome, perhaps to 200–400 positional candidate genes. Locating the gene(s) responsible and identifying the causal molecular genetic variation is a major challenge. Nevertheless, there have been some striking successes in achieving this goal in pigs, to which some of the co-authors have contributed.

The only limitation to performing direct genetic experiments and identifying genes underlying these traits is the lack of a complete genome sequence. Selection experiments, heterosis studies and breed comparisons have all been used in porcine genetic studies. Many populations have been used to map genes to large chromosomal regions but positional mapping of causal genes has been difficult. Sequencing the porcine genome and generating 100 000 SNPs will provide additional polymorphic markers and positional candidate genes based on the human and mouse map. Large populations with designed matings can be used to positionally map genes. The populations can be generated by natural reproduction, artificial insemination or assisted reproductive technologies. Clones can also be generated from fibroblasts or stem cells and

Copyright © 2005 John Wiley & Sons, Ltd.

cryopreserved. This technology provides the opportunity for knock-out or knock-in experiments in an animal other than the mouse. Interspecies porcine hybrids are easily produced and are very valuable for knock-out/knock-in experiments and studying genomic imprinting (Andersson *et al.*, 1994).

### Justification for sequence information

A CREES-USDA workshop during the summer of 2002, The Allerton III Conference ('Beyond Livestock Genomics') was designed to bring together leading investigators from broad disciplines (physiology, reproduction, animal health, nutrition and genetics) to begin to develop a plan for full utilization of genomic information to promote animal health and production (Hamernik *et al.*, 2003). In February 2002, the National Academy of Sciences organized a public workshop, 'Exploring Horizons for Domestic Animal Genomics', to identify research goals and funding needs. Subsequent discussion identified a growing need to have a broader context for discussion to ensure full utilization of the genomic information and tools in support of animal research. Thus, the Allerton III Conference provided a venue for discussion of how genome sequences could be harvested to support the broader animal agricultural community, while contributing to life science discovery. The objectives of the Allerton III Conference included: (a) identification of genomic and bioinformatic tools and reagents required to exploit information from the human genome initiative; (b) discussion of needs and opportunities for full implementation of genomic capabilities by related disciplines; and (c) identification of needs and opportunities to ensure full technology transfer and commercialization (Hamernik *et al.*, 2003).

### The Swine Genome Sequencing Consortium (SGSC)

In September 2003, interested researchers convened at INRA-Jouy-en-Josas to establish the SGSC for facilitation and coordination of international efforts toward obtaining the complete porcine genome sequence. A coordinated international effort was initiated to develop a porcine BAC map with two BAC libraries (RPCI-44 and CHORI-242) made by Pieter J. de Jong, one library made at the Roslin Institute (Anderson *et al.*, 2000), and a library produced at INRA (Rogel–Gaillard *et al.*, 1999). Through the exchange of BAC clones, data has been merged to permit a comprehensive analysis. INRA has screened more than 1000 BACs from this library for known genes and markers and has mapped them on genetic and RH maps. INRA is sharing this set of BACs to facilitate anchoring of contigs. Sequencing the ends of all fingerprinted BAC clones has also been conducted. The current status of the fingerprint contig (FPC) was discussed at the PAG 2005 meeting (Humphray *et al.*, 2005; see Table 1). The final product, which is scheduled for completion in July 2005, will represent 20× coverage of the porcine genome.

During the past year, significant allocation of resources has occurred with respect to positioning the porcine genome sequencing initiative. This has included the development of a whole genome porcine BAC fingerprint with complete BAC end-sequencing. Thus, to date, the SGSC has completed sequencing of over 500 000 BAC ends (see Table 2), which represents over 13% sequence coverage of the pig genome (Humphray *et al.*, 2005).

**Table 1.** FPC Database (Wellcome Trust Sanger Institute)

| Library | Fingerprints | Complexity[c] |
|---|---|---|
| CHORI-242 | 103 762[a] | 6.3× |
| PigE BAC (Roslin) | 73 971[a] | 4.0× |
| RPCI-44 | 61 104[b] | 4.0× |
| INRA | 28 478[a,b] | 1.0× |
| TOTAL | 267 826 | 15.3× |

[a] Wellcome Trust Sanger Institute: **http://www.sanger.ac.ak/Projects/S_scrofa/**
[b] University of Illinois at Urbana-Champaign.
[c] Based on 2.7 Gb genome size.

**Table 2.** BAC end sequencing results

| Library | Passed reads | Paired ends (%) | Average GC (%) | Average length (bp) |
|---|---|---|---|---|
| CHORI-242[a,b] | 276 758 | 92 | 41 | 698 |
| PigE BAC[a] | 145 110 | 93 | 42 | 700 |
| RPCI-44[b] | 71 847 | 87 | 40 | 521 |
| INRA[c] | 64 102 | 94 | 42 | 613 |
| Total | 557 898 | BACs representing 13% of pig genome | | |

[a] Wellcome Trust Sanger.
[b] University of Illinois at Urbana–Champaign.
[c] INRA.

Copyright © 2005 John Wiley & Sons, Ltd. 

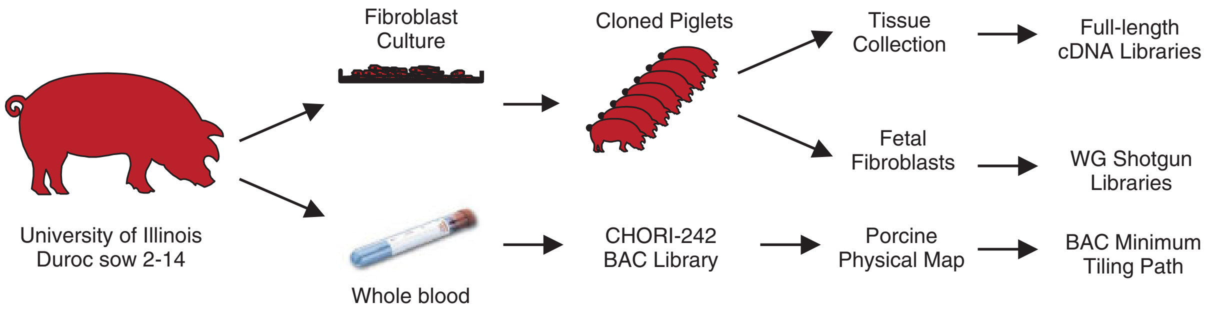

**Figure 1.** Schema for the production of autologous reagents for sequencing the porcine genome. Duroc sow 2–14 was selected as the donor of the DNA used to create the CHORI-242 BAC library. She has been 'immortalized' by the establishment of fibroblast cell cultures that have been successfully used for nuclear transfer. Fetal fibroblast cultures from cloned piglets have been transferred to the Sanger Institute and genomic DNA from these cell lines has been used to create whole-genome shotgun libraries. Tissues from embryos at various stages of gestation have also been collected for use in the construction of full-length cDNA libraries

### The sequencing template

The majority of clones that have been fingerprinted and end-sequenced have come from the CHORI-242 BAC library. This library was constructed from a single female pig that was raised at the University of Illinois (Figure 1). To facilitate sequence assembly, efforts will be made to select as many CHORI-242 clones as possible for the BAC minimum tiling path. Additionally, the WGS libraries will be made from autologous DNA to further enhance sequence assembly between WGS reads and those from the BAC skim. Full-length cDNA libraries will also be constructed from tissues belonging to the original sow or her clones, providing autologous sequence for gene annotation.

### Strategy for genomic sequencing

The strategy that we espouse for sequencing the pig genome combines the whole-genome shotgun (WGS) approach with skim sequencing of BAC clones selected to represent a minimum tiling path through the pig genome (Engler *et al.*, 2003; She *et al.*, 2004). We propose that a draft sequence of the pig genome with 6–7× genome coverage be produced by this hybrid approach. A draft sequence does not provide complete coverage of the entire genome; indeed, there are still gaps in the current 'finished' human genome sequence. One of the key strengths of the hybrid approach is that the resources (BAC clones) will be in place for targeted sequence closure in regions of interest. An important difference between the application of this approach to the pig genome and its use for other species to date is that the porcine fingerprint map and BAC end sequence information will be completed before the sequencing project starts. Thus, it should be possible to determine a BAC tiling path from these two datasets, identifying a set of BACs with minimal overlap at the outset of the sequencing project. Current calculations predict that at most 25 000 BACs will need to be sequence-skimmed, since the human genome is approximately 2.9 GB and the pig genome is approximately 2.6 GB. This calculation is also supported by the increased size of the BAC inserts from 150 kb to a range of 160–180 kb, thus reducing the number of BACs to be sequence-skimmed. The project will then sequence 3× coverage and the remaining 3–4× coverage will come from whole-genome sequencing of 3 kb, 10 kb and 50 kb libraries. The sequence will be released into public databases as it is generated, and sequence traces will be deposited in the trace repositories hosted at NCBI and EBI. Sequence assemblies >2 kb will be deposited in the HTGS databases at NCBI and EMBL. It is anticipated that after the first year of sequencing, a draft 3× assembly of the genome will be released into public databases.

### Acknowledgements

This work was partially supported by grants from the USDA-National Research Initiative (2002-35205-12712), the USDA Cooperative State Research Service (AG2002-34480-11828) and the USDA Agricultural Research Service (Agreement No. 58-5438-2-313). The authors wish

Copyright © 2005 John Wiley & Sons, Ltd.

to recognize the support of the College of Agricultural, Environmental and Consumer Sciences, University of Illinois. The SGSC also wishes to recognize the leadership provided by USDA Undersecretary J. Jen (in promoting animal genomics) and the CSREES (C. Hefferan, A. Palamiso, M. Poth, D. Hamernik, M. Qureshi, P. Brayton) and ARS (R. Green and S. Kappes) administration, and the Alliance for Animal Genome Research (R. Wyse and K. Eversole). Finally, support and assistance from M. Boggess of the NPB is acknowledged. Also, support from the UK BBSRC, Defra, Sygen and Roslin Institute is acknowledged.

### References

Anderson SI, Lopez-Corrales NL, Gorick B, Archibald AL. 2000. A large fragment porcine genomic library resource in a BAC vector. *Mamm Genome* **11**: 811–814.

Andersson L, Haley CS, Ellegren H, *et al*. 1994. Genetic mapping of quantitative trait loci for growth and fatness in pigs. *Science* **263**: 1771–1774.

Archibald AL, Haley CS, Brown JF, *et al*. 1995. The PiGMaP Consortium linkage map of the pig (*Sus scrofa*). *Mamm Genome* **6**: 157–175.

Bidanel JP, Rothschild MF. 2002. Current status of quantitative trait loci mapping in pigs. *Pig News Inform* **23**: N39–N54.

Chowdhary BP, Raudsepp T, Fronicke L, Scherthan H. 1998. Emerging patterns of comparative genome organization in some mammalian species as revealed by ZOO-FISH. *Genome Res* **8**: 577–589.

Ellergren H, Chowdhary BP, Johansson M, *et al*. 1994. A primary linkage map of the porcine genome reveals a low rate of genetic recombination. *Genetics* **137**: 1089–1100.

Engler FW, Hatfield J, Nelson W, Soderlund CA. 2003. Locating sequence on FPC maps and selecting a minimal tiling path. *Genome Res* **13**: 2152–2163.

Fahrenkrug SC, Rohrer GA, Freking BA, *et al*. 2001. A porcine BAC library with tenfold genome coverage: a resource for physical and genetic map integration. *Mamm Genome* **12**: 472–474.

Goureau A, Yerle M, Schmitz A, *et al*. 1996. Human and porcine correspondence of chromosome segments using bidirectional chromosome painting. *Genomics* **36**: 252–262.

Gregory SG, Sekhorn M, Schein J, *et al*. 2002. A physical map of the mouse genome. *Nature* **418**: 743–749.

Hamernik DL, Lewin HA, Schook LB. 2003. Allerton III. Beyond Livestock Genomics. *Anim Biotech* **14**: 77–82.

Hawken RJ, Murtaugh J, Flickinger GH, *et al*. 1999. A first generation porcine whole-genome radiation hybrid map. *Mamm Genome* **10**: 824–830.

Humphray SJ, Clark RC, Beever J, *et al*. 2005. An integrated physical map of the porcine genome. Proceedings of the Plant and Animal Genome XIII Conference, San Diego, CA: abstr P559; 210.

Malek M, Dekkers JCM, Lee HK, Baas TJ, Rothschild MF. 2001a. A molecular genome scan analysis to identify chromosomal region influencing economic traits in the pig. I. Growth and body composition. *Mamm Genome* **12**: 630–636.

Malek M, Dekkers JCM, Lee HK, *et al*. 2001b. A molecular genome scan analysis to identify chromosomal regions influencing economic traits in the pig. II. Meat and muscle composition. *Mamm Genome* **12**: 630–636.

Maruyama K, Sugano S. 1994. Oligo-capping: a simple method to replace the cap structure of eukaryotic mRNAs with oligoribonucleotides. *Gene* **138**: 171–174.

Meyers SN, Rogatcheva MB, Yerle M, *et al*. 2005. Piggy–BACing the human genome: II. A high resolution, physically-anchored, comparative map of the porcine autosomes. *Genomics* (in press).

Milan D, Jeon TT, Looft C, *et al*. 2000. A mutation in PRKAG3 associated with excess glycogen content in pig skeletal muscle. *Science* **288**: 1248–1251.

Nezer C, Moreau L, Wagenaar D, Georges M. 2002. Results of a whole-genome scan targeting QTL for growth and carcass traits in a Pietrain X Large White intercross. *Genet Select Evol* **34**: 371–387.

Paszek AA, Wilkie PJ, Flickinger GH, *et al*. 1999. Interval mapping of growth in divergent swine cross. *Mamm Genome* **10**(2): 117–122.

Rettenberger G, Klett C, Zecher U, *et al*. 1995. Visualization of the conservation of synteny between humans and pigs by heterologous chromosomal painting. *Genomics* **26**: 372–378.

Rink A, Santchi EM, Eyer KM, *et al*. 2002. A first-generation EST RH comparative map of the porcine and human genome. *Mamm Genome* **13**: 578–587.

Rohrer GA, Alexander LJ, Hu Z, *et al*. 1996. A comprehensive map of the porcine genome. *Genome Res* **6**: 371–391.

Rohrer GA, Ford JJ, Wise TH, Vallet JL, Christenson RK. 1999. Identification of quantitative trait loci affecting female reproduction traits in a multigeneration Meishan–White composite swine population. *J Anim Sci* **77**: 1385–1391.

Rogel-Gaillard C, Bourgeaux N, Billaut A, Vaiman M, Chardon P. 1999. Construction of a swine BAC library: application to the characterization and mapping of porcine type C endoviral elements. *Cytogenet Cell Genet* **85**: 205–211.

She XZ, Jiang RA, Clark G, *et al*. 2004. Shotgun sequence assembly and recent segmental duplications within the human genome. *Nature* **431**: 927–930.

Thomas JW, Touchman JW, Blakesley RW, *et al*. 2003. Comparative analyses of multi-species sequences from targeted genomic regions. *Nature* **424**: 788–793.

Wilkie PJ, Paszek AA, Beattie CW, *et al*. 1999. A genomic scan of porcine reproductive traits reveals a possible quantitative trait loci (QTL) for number of corpora lutea. *Mamm Genome* **10**: 573–578.

Yerle M, Echard G, Robic A, *et al*. 1996. A somatic cell hybrid panel for pig regional gene mapping characterized by molecular cytogenetics. *Cytogenet Cell Genet* **73**: 194–202.

Yerle M, Pinton P, Delcros C, *et al*. 2002. Generation and characterization of a 12 000-rad radiation hybrid panel for fine mapping in pig. *Cytogenet Genome Res* **97**: 219–228.

Copyright © 2005 John Wiley & Sons, Ltd. 
